# Semicircular canal biomechanics in health and disease

**DOI:** 10.1152/jn.00708.2018

**Published:** 2018-12-19

**Authors:** R. D. Rabbitt

**Affiliations:** ^1^Department of Biomedical Engineering, University of Utah, Salt Lake City, Utah; ^2^Otolaryngology-Head Neck Surgery, University of Utah, Salt Lake City, Utah; ^3^Neuroscience Program, University of Utah, Salt Lake City, Utah

**Keywords:** alcohol nystagmus, benign paroxysmal positional vertigo, canal dehiscence, caloric, crista ampullaris, inner ear, labyrinth, Ménière’s disease, motion sensation, neural encoding of movement, Tullio phenomena, vestibular

## Abstract

The semicircular canals are responsible for sensing angular head motion in three-dimensional space and for providing neural inputs to the central nervous system (CNS) essential for agile mobility, stable vision, and autonomic control of the cardiovascular and other gravity-sensitive systems. Sensation relies on fluid mechanics within the labyrinth to selectively convert angular head acceleration into sensory hair bundle displacements in each of three inner ear sensory organs. Canal afferent neurons encode the direction and time course of head movements over a broad range of movement frequencies and amplitudes. Disorders altering canal mechanics result in pathological inputs to the CNS, often leading to debilitating symptoms. Vestibular disorders and conditions with mechanical substrates include benign paroxysmal positional nystagmus, direction-changing positional nystagmus, alcohol positional nystagmus, caloric nystagmus, Tullio phenomena, and others. Here, the mechanics of angular motion transduction and how it contributes to neural encoding by the semicircular canals is reviewed in both health and disease.

## INTRODUCTION

**Fig. 1.**
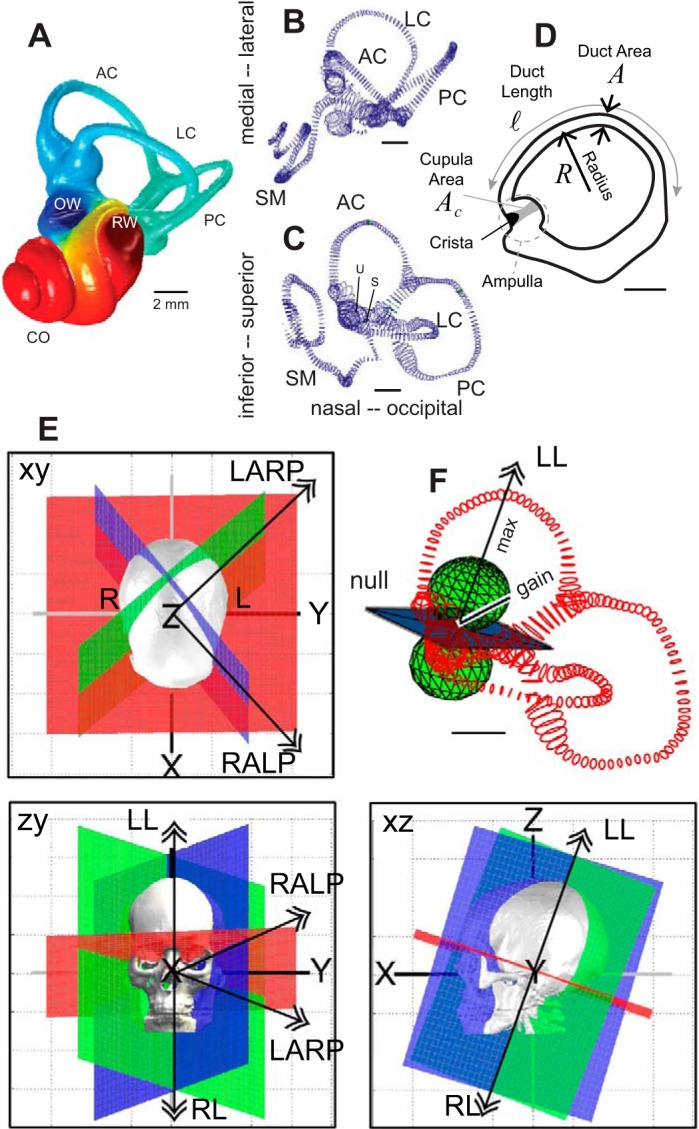
Human vestibular labyrinth. *A*: reconstruction of a human osseous labyrinth showing the gross morphology of the bony cavity occupied by the anterior semicircular canal (AC; blue), lateral canal (LC), posterior canal (PC; teal), and the cochlea (CO; red). The locations of the oval window (OW) and round window (RW) are indicated. *B* and *C*: adult human membranous labyrinth showing the endolymph-filled LC, AC, PC, cochlear scala media (SM), and locations of the utriculus (U) and sacculus (S) from 2 orthographic views. *D*: outline of the LC membranous labyrinth projected in the plane of the canal showing the location of the ampulla, cupula, crista, and cupula. Key dimensions are indicated in italics. *E*: anatomical canal planes determined from human computed tomography scans annotated with double arrows showing right-hand-rule rotation directions that maximize excitation of the left AC and right PC (LARP), right AC and left PC (RALP), right LC (RL), and left LC (LL). *F*: the mechanical gain is zero for rotation in the null plane and maximum for rotation in directions perpendicular to the null plane, shown for the LC (LL, double arrow). Sensitivity obeys the 3-dimensional cosine rule, with the magnitude of the mechanical response denoted by the distance from the pole of the sphere to the surface of the sphere (gain). [*A*: provided by Iversen et al. (2016); *B*–*D*: adapted from Ifediba et al. (2007); and *E*: adapted from Della Santina et al. (2005).]

The inner ear vestibular organs are phylogenetically ancient mechanosensory transducers that provide amniotes with the ability to sense movement and orientation of the head relative to gravity. The complete vestibular apparatus first appeared in primitive fish, predating the appearance of the mammalian cochlea by hundreds of millions of years (Fritzsch 1998; Luo et al. 2011; Northcutt and Gans 1983; Stensiö 1927). Figure 1, *A*–*C*, shows the gross morphology of the human labyrinth, where each ear includes two otolith organs responsible for sensing linear motion and gravity (Fig. 1*C*, utriculus and sacculus) and three semicircular canals responsible for sensing angular motion [lateral (Fig. 1*A*), anterior (Fig. 1*B*), and posterior canals (Fig. 1*C*)]. In some species these five primary vestibular organs are augmented by a papilla neglecta that supplements angular motion sensation by the canals (Brichta and Goldberg 1998) and by a lagena that supplements function of the otolith organs and can play a role in magnetoreception (Fritzsch et al. 1990; Khorevin 2008; Lu et al. 2003; Wu and Dickman 2011; Zakir et al. 2012). Although there are dramatic differences in gross morphology between hearing organs across vertebrate classes, the vestibular semicircular canals and the utriculus are largely conserved with differences primarily in dimensions and fine structure (Araujo et al. 2018; Blanks et al. 1985; Curthoys and Oman 1987; Ekdale 2013; Engstrom et al. 1966; Ewald 1892; Ghanem et al. 1998; Goltz 1870; Igarashi 1966; Igarashi et al. 1981; Schmelzle et al. 2007; Spoor et al. 2007; Wersäll and Bagger-Sjöbäck 1974). As described in more detail below, endolymph displacement within each canal in response to angular head acceleration is explicitly dependent on key morphological features including the radius of the canal (*R*), the length of the slender duct (*l*), the cross-sectional area of the slender duct (*A*), the cross-sectional area (*A*_c_) and thickness of the cupula (*h*), and the orientation of the canal plane relative to the direction of head rotation (Fig. 1, *D*–*F*). Dependence on morphology allows straightforward comparison of mechanics across species. Interspecies morphological homology does not extend directly to auditory or mixed auditory/vestibular organs. In fish, for example, the vestibular sacculus serves as the primary hearing organ and provided ancient fish with the ability to detect sound and vibration well before the appearance of more specialized sensory hair cell hearing organs in reptiles, amphibians, birds, and mammals (Dooling et al. 2000). Relatively large interspecies differences in saccular morphology are present in fish (Deng et al. 2011; Schulz-Mirbach et al. 2011a, 2011b), specializations likely driven by selective pressure to detect specific auditory frequencies favoring survival of the species. In modern mammals, both the sacculus and the utriculus retain the ability to sense auditory frequency sound and vibration (Curthoys et al. 2016; McCue and Guinan 1994; Young et al. 1977), but their role in auditory sensation has been largely supplanted by the mammalian cochlea, which dramatically extends the frequency bandwidth and dynamic range of hearing relative to what can be achieved by otolith organs.

The intact semicircular canals are highly selective to sense angular motion (Breuer 1874; Camis 1930; Flourens 1824; Goldberg et al. 2012; Mach 1874). Directional selectivity, sensitivity, speed, and dynamic range are dependent on biomechanics of the apparatus, which prescribes the relationship between angular head accelerations in three-dimensional space and microscale sensory hair bundle displacements. This review describes the role of semicircular canal biomechanics in angular motion sensation and how common disorders altering the biomechanics can lead to transmission of inappropriate signals to the central nervous system (CNS).

## MECHANICS OF THE SEMICIRCULAR CANALS IN HEALTH

### Physical and Biological Properties

The semicircular canals are housed in a rigid labyrinthine cavity located in the temporal bone (Fig. 1*A*). The gross structure consists of three toroidal loops that are responsible for angular motion sensitivity and canal-specific directional selectivity. Inside the bony cavity is a membranous labyrinth (Fig. 1, *B* and *C*) that conforms to the complex shape and gross dimensions of the bony labyrinth. The primary role of the membranous labyrinth is to provide the electrochemical barrier separating K^+^-rich endolymph inside the membrane from Na^+^-rich perilymph outside the membrane (Anniko and Wroblewski 1980), a barrier essential to sensory hair cell function in both the cochlea and vestibular organs (Mittal et al. 2017; Nin et al. 2016; Salt and Hirose 2018). The difference in ionic concentrations between the two inner ear fluids generates a large resting endolymphatic potential in the cochlea of approximately +80 mV and approximately +10 mV in semicircular canal ampullae (Highstein et al. 1996; Köppl et al. 2018; Salt and Konishi 1982; Wangemann 2002). Some reports suggest the semicircular canal endocupular potential exceeds the endolymphatic potential in ampullae and could be as high as +70 mV based on microelectrode measurements (Köppl et al. 2018; Trincker 1957), but reversal of the microphonic suggests the voltage acting across the apical surface of hair cells might be closer to the endolymphatic potential of +10 mV at least in some species (Rabbitt et al. 2005). Ionic compositions determine the electrochemical potential responsible for driving the mechanoelectrical transduction (MET) current into sensory hair cells. Both fluids are approximately Newtonian with viscosity (μ) and density (ρ) similar to water (Steer et al. 1967). The endolymph has high macromolecular content (Thalmann et al. 1992, 2006; Thalmann and Thalmann 1999), implying its density and rheological properties might be sensitive to pathological or experimental conditions if its molecular composition or state are altered. The membranous labyrinth separating the two fluids consists of a thin extracellular matrix lined on the inside lumen by a single monolayer of epithelial cells (~5-µm thick). The extracellular matrix is bathed in perilymph and is responsible for the mechanical integrity of the membranous labyrinth. This structure varies in thickness between species and location in the labyrinth (~15- to 200-µm thick) and is largely acellular. The monolayer of extrasensory epithelial cells provides the electrochemical barrier between endolymph and perilymph (Köppl et al. 2018). Compromise of the permeability or transport function of this epithelial barrier would be expected to result in loss of hair cell and sensory organ function (Kim and Marcus 2009; Kitajiri and Katsuno 2016; Runggaldier et al. 2017; Sun and Wang 2015).

**Fig. 2.**
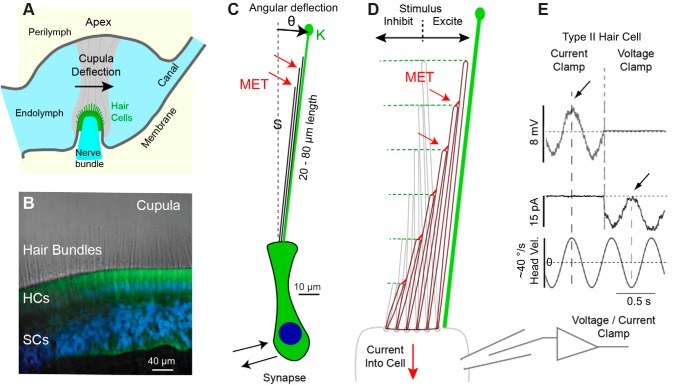
Mechanotransduction. *A*: schematic showing the cupula (gray) spanning the entire lumen of the ampulla and enveloping hair bundles at the surface of the sensory epithelium. *B*: hair cell (HC) stereocilia project as stiff actin core rods 20–80 µm into the cupula (toadfish crista. Green: xyloside (BX) in HCs and supporting cells (SCs). Blue: DAPI nuclear stain. *C*: schematic of type I murine HC and bundle illustrating the glycocalyx (green) around the kinocilia (K) that integrates with the cupula, and putative locations of mechanoelectrical transduction channels (MET; red arrows) at the tips of stereocilia (S) 20–80 µm above the HC body (sketch to scale). *D*: stereocilia are stiff and deflect almost as rigid rods pivoting at their base. Deflection toward the kinocilia increases the transduction current and is excitatory, while deflection away decreases the transduction current and is inhibitory (aspect ratio not to scale, bundles are longer than depicted). *E*: current- and voltage-clamp recordings of semicircular canal HC in vivo demonstrate the MET current closely follows bundle displacement for small physiological stimuli. Nonlinearities arise at higher stimulus levels. [*B*: provided by Holman et al. (2016); and *E*: adapted from Rabbitt et al. (2005).]

Conversion of endolymph displacement into neural signals takes place in an enlarged region of each canal called the ampulla where fluid displacement generated by angular head acceleration deflects the cupula and activates sensory hair cells. The cupula is a glycosaminoglycan (GaG)-rich structure that completely spans the cross section of the ampulla and couples motion of the endolymph to deflection of sensory hair bundles (Fig. 2*A*) (Dernedde et al. 2014; Dohlman 1981; Gioglio et al. 1995; Köppl et al. 2018; Silver et al. 1998; Sugiyama et al. 1991; Takumida et al. 1989). Under normal physiological conditions, the cupula is completely attached around its periphery and precludes the movement of endolymph from one side of the ampulla to the other (Hillman 1974; McLaren and Hillman 1979; Rabbitt et al. 2009). The maximum deflection of the cupula for physiological head movements occurs near its center and is predicted on the basis of direct measurements in animals to have a magnitude on the order of micrometers in humans for stimuli in the volitional range of head movements (Oman et al. 1979; Rabbitt et al. 2009, 2010; Selva et al. 2009). Cupula displacements are very small relative to its dimensions, so the mechanical deformation gradient inside the cupula is small (e.g., the Lagrangian strain is small). Since the endolymph cannot pass unimpeded through or by the intact cupula, there is no true convective fluid flow around the canal in the healthy ear but only small fluid particle displacements away from their resting position.

Mechanosensitive hair cells are located on the apical surface of the crista in a thin sensory epithelium. Motion of the cupula is detected by displacement of specialized microvilli called stereocilia projecting from the apical surface of hair cells into the cupula (Fig. 2*B*, example from toadfish). Sensory hair bundles extend from the apical face of hair cells 20–80 µm into the cupula and are extremely long relative to sensory hair cells in other organs including the cochlea, utricle and saccule (Fig. 2*C*, sketch of type 1 hair cell from the mouse crista shown to scale). Each bundle consists of a single tall kinocilium (Fig. 2*C*, kinocilia) flanked by a steep array of stereocilia on one side (Fig. 2*C*, stereocilia). Several stereocilia within the bundle can approach the length of the kinocilium, but others are relatively short and project only partially up the bundle (Flock and Orman 1983; Harada 1972; Lim 1971). The kinocilium is enveloped by a glycocalyx that extends into the cupula and provides a mechanical link putatively forcing deflection of the kinocilium to follow the cupula (Holman et al. 2016). There is evidence that strong coupling of kinocilium to the overlying accessory structures requires the proteins otogelin (OTOG gene) and α-Tectorin (TECTA gene), mutations of which can cause deafness and vestibular dysfunction (Stooke-Vaughan et al. 2015). Direct coupling between the kinocilium and the cupula is likely to be a mechanical factor needed for semicircular canal afferent neurons to detect low-frequency or static stimuli over very long periods of time.

Stereocilium within the bundles contain exquisitely sensitive MET (Fig. 2, *C* and *D*) channels responsible for sensing displacement of the bundle (Fritzsch and Straka 2014; Hudspeth 1985; Straka and Baker 2013). Deflection of the hair bundle toward the kinocilium opens MET channels at the tips of stereocilia leading to the influx of K^+^ and Ca^+2^ into the hair cell (Beurg et al. 2009; Hudspeth 1982; Lumpkin and Hudspeth 1995). Cation influx depolarizes the cell, leads to excitatory synaptic transmission, and modulates the discharge rate of afferent neurons (Fettiplace 2017; Moser et al. 2006). Optical images of long sensory hair bundles in the frog crista after digestion of the cupula demonstrate bundles are “stiff,” “pivot around their base,” and “move together as if joined” (Flock and Cheung 1977; Flock et al. 1977). Splay between individual stereocilia in canal hair cells generated by physiological stimulation, if any, has not yet been investigated. Stereocilium stiffness arises from the dense crosslinked actin core, which tapers at the rootlet to allow pivoting at the base (Flock and Cheung 1977; Francis et al. 2015; Krey et al. 2017). This is illustrated schematically in Fig. 2*D* (aspect ratio not to scale) where the bundle is deflected in the excitatory direction leading to the opening of MET channels. Based on data from other inner ear organs, MET channels in crista hair cells are likely located at the tips of stereocilia (Beurg et al. 2009; Lumpkin and Hudspeth 1995) and gated by extracellular filaments composed of cadherin-23 and protocadherin-15 spanning between stereocilia (Kazmierczak et al. 2007; Sakaguchi et al. 2009). Transmembrane proteins from the transmembrane channel-like family have been identified as the most likely MET channel candidates, but other proteins are also involved (Kurima et al. 2015; Xiong et al. 2012; Zhao et al. 2014). It is not yet known if mechanosensitive *Piezo* channels located on the apical surface of auditory hair cells (Wu et al. 2017) might augment MET channels in the stereocilia or play a role in crista hair cell mechanotransduction. Angular deflection of the bundle (θ, radians) pivoting about the base generates relative shear displacement between adjacent stereocilia that is responsible for mechanical gating of MET channels in stereocilia (Howard and Hudspeth 1988; Markin and Hudspeth 1995). Based on measurements in toadfish, the deflection of the cupula is approximately ±1 µm in response to sinusoidal head oscillations delivered with a peak angular velocity of ±10 °/s (Rabbitt et al. 2009, 2010). Although not yet measured directly in vivo, a cupula displacement of ±1 µm would be expected to generate angular hair bundle displacements less than ±0.01 rad. Displacements of this magnitude are quite small relative to the operating range of the MET apparatus (Eatock et al. 2006; Fettiplace 2017), thereby allowing the MET current in canal hair cells to modulate in both excitatory and inhibitory directions relative to the resting state. This is illustrated in Fig. 2*E* by current-clamp and voltage-clamp recordings from a type II semicircular canal hair cell in response to 2-Hz stimulus (data from toadfish in vivo). The ability to respond in both inhibitory and excitatory directions for physiological stimuli putatively underlies the nearly symmetric responses observed in a subset of regularly discharging semicircular canal afferent neurons as discussed below. The stimulus used in Fig. 2*E* would be expected to generate approximately ±2 µm sinusoidal deflection of the cupula and approximately ±0.02 rad angular displacement of hair bundles. Larger hair bundle deflections (e.g., ±0.1 rad) can saturate the MET channels leading to saturated responses and larger excitatory-inhibitory nonlinearity.

### Coding of Angular Motion

The neural code transmitted to the CNS by semicircular canals is complex, with individual afferent neurons within each nerve bundle exhibiting diverse kinetics. In this section, adaptive properties of semicircular canal afferents are briefly described to illustrate what can be explained by mechanics and what cannot.

**Fig. 3.**
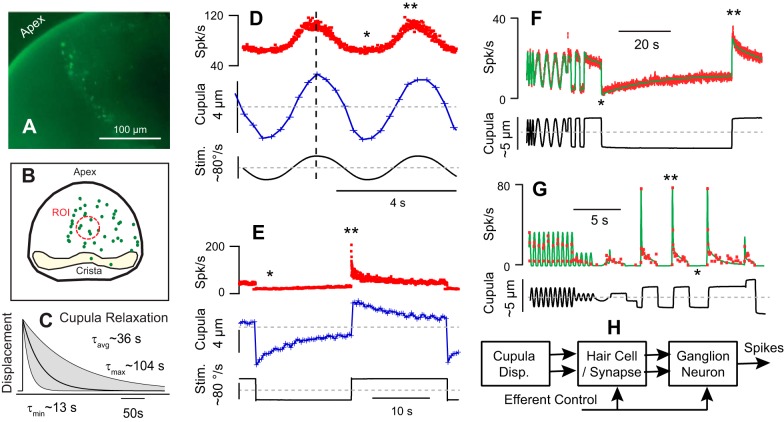
Cupula displacement vs. afferent response. *A*: image of fluorescent beads adhered to the cupula in the toadfish animal model. *B*: reconstruction of bead locations highlighting the region of interest where bead motion was tracked. *C*: the time constant of cupula. Mechanical relaxation averaged 36 s (13–104 s range) for step velocity stimuli, considerably slower than the average adaptation time constant recorded in afferent neurons for the same stimulus. *D*: Simultaneous recordings of afferent nerve action potential rate (red) and cupula displacement (blue) for a low-frequency sinusoidal stimulus. The stimulus was mechanical indentation of the canal duct about a preload, mimicking sinusoidal angular velocity stimuli at ~40°/s zero-to-peak (Dickman et al. 1988; Rabbitt et al. 1995). *E*: second example showing mechanical relaxation of the cupula (blue) and nonlinear adaptation of an example afferent (red) for a stimulus equivalent to ~80°/s step in angular velocity. Sensitivity and adaptation are both higher for excitatory stimuli relative to inhibitory stimuli. *F*: example afferent (red) that follows displacement of the cupula with weak inhibitory-excitatory nonlinearity and only modest adaptation. *G*: example afferent with inhibitory cut off and rapid adaptation following 2 time constants. *F* and *G*: solid curves (green) are predictions of simple mathematical model of nonlinear afferent discharge. *H*: major biophysical stages of signal processing leading to modulation of afferent neuron action potentials. *Inhibitory nonlinearity. **Rapid excitatory nonlinearity. [*A*–*C* adapted from Rabbitt et al. (2009); *D* and *E* from Rabbitt et al. (2010); and *F* and *G*: from Rabbitt et al. (2005).]

The seminal work of J. Goldberg and C. Fernandez initiated in the early 1970s details semicircular canal afferent nerve responses to angular motion stimuli in mammals (Baird et al. 1988; Curthoys et al. 1995; Fernandez et al. 1972, 1988, 1990, 1995; Fernandez and Goldberg 1971, 1976; Goldberg et al. 1985, 1990a, 1990b; Goldberg and Fernandez 1971a, 1975, 1980; Young et al. 1977). Although there are some differences in sensitivity and dynamics, neural encoding of angular head movements shows strong similarities across extant species (Blanks and Precht 1976; Boyle and Highstein 1990; Goldberg et al. 2012; Honrubia et al. 1989; Hullar et al. 2005; Kim et al. 2011; Lambert et al. 2008; Lasker et al. 2008; Paulin and Hoffman 1999; Ramachandran and Lisberger 2006; Sadeghi et al. 2007b). In all cases, encoding varies between individual afferent neurons within nerve branches arising from each ampulla, with some units modulating their action potential discharge rate in direct proportion to angular head velocity in the sensitive canal plane over a broad range of oscillatory head frequencies, and other units exhibiting frequency-dependent sensitivity and timing of peak discharge relative to the stimulus. The origin of this diversity arises primarily from morphophysiology and cellular biophysics of hair-cell/afferent complexes rather than diversity in macromechanical hair bundle inputs. The signal processing is highlighted in Fig. 3 where the displacement of fluorescent beads attached to the cupula was compared directly to afferent nerve discharge in the toadfish. Since the toadfish gross morphology, size, and structure are very similar to adult humans, cupula displacement would be expected to be similar. This animal model also facilitates both mechanical and electrophysiological recordings and may be the only species to date where cupula motion and afferent responses have been recorded simultaneously in vivo (Rabbitt et al. 2009, 2010). Displacement of the cupula depends on macromechanics and exhibits a simple relaxation behavior, while temporal modulation of afferent neurons is more complex and diverse across individual units. This diversity in signal processing is responsible for parsing angular head movement signals into parallel channels encoding various aspects of the stimulus and transmitted simultaneously to the CNS (Sadeghi et al. 2007a).

The mechanical relaxation time constant of the cupula can be observed directly using step stimuli. A step increase in angular velocity, or a step mechanical indentation of the slender duct, causes a very rapid displacement of the cupula that is followed by a period of relaxation when the cupula returns to its resting position (Fig. 3*C*). The relaxation time constant in has been reported to range from 13 to 104 s, measured in toadfish (Rabbitt et al. 2009). As described below, this mechanical time constant results from a balance between the cupula elasticity that restores the resting position of the cupula and the endolymph viscosity that resists fluid motion. For physiological stimuli the cupula response is nearly linear, exhibiting only a small nonlinearity at very low stimulus levels (Rabbitt et al. 2010). This small nonlinearity shows similarity to hair bundle nonlinearity observed in other inner ear organs and likely has origins in an active process associated with the mechanoelectrical transduction machinery (Fettiplace 2017; Martin and Hudspeth 1999; Nin et al. 2012).

Direct comparison of cupula displacement to action potential discharge rates demonstrates that some afferent neurons directly encode macromechanical displacement of the cupula, while other units show extensive adaptation and excitatory-inhibitory nonlinearity not present in the mechanics. This is illustrated in Fig. 3, which compares afferent responses to cupula displacement, both measured simultaneously in the toadfish. The afferent in Fig. 3*D* (red, spike/s), for example, closely follows cupula displacement (blue, µm) and the low-frequency sinusoidal stimulus (black, º/s), with the exception of a modest reduction in sensitivity for inhibitory stimuli. The afferent in Fig. 3*E*, in contrast, shows a strong excitatory-inhibitory nonlinearity and strong adaptation during the excitatory phase of the step stimulus, absent during inhibition. Figure 3, *F* and *G*, provides two additional examples of afferent discharge rate highlighting the large differences in between individual units, with Fig. 3*F* exhibiting very little adaptation for a 60-s step stimulus vs. Fig. 3*G* exhibiting rapid adaptation and complete inhibitory saturation silencing the unit. Data in Fig. 3, *F* and *G*, were obtained by forcing the displacement of the cupula in a prescribed manner and hence do not include mechanical relaxation of the cupula, while data in Fig. 3, *D* and *E*, were obtained using stimuli mimicking natural angular head velocity and therefore include mechanical relaxation of the cupula. The units shown in Fig. 3, *D* and *F*, from toadfish have modestly adapting response properties similar to regularly discharging afferents in mammals, while the units shown in Fig. 3, *E* and *G*, have strong adaptation similar irregularly discharging afferents in mammals (Fernandez and Goldberg 1971; Goldberg and Fernandez 1971b). Postmechanical signal processing is responsible for this diversity and arises from diversity in hair cells, synapses, and afferent neurons simplified to a the block diagram in Fig. 3*H* (Eatock et al. 2008; Glowatzki and Fuchs 2002; Goldberg et al. 2000, 2012; Highstein et al. 2015; Kalluri et al. 2010; Perez et al. 2010; Sadeghi et al. 2007a, 2014; Songer and Eatock 2013).

Action potentials transmitted to the CNS from the canals encode both the direction and the time course of angular head movements. Directional coding arises from the nearly orthogonal orientation of the canals, which maximizes sensitivity of each canal to a specific direction of head rotation, thereby decomposing three-dimensional (3D) head rotations into three vector components, one transmitted by each individual nerve branch innervating each crista. This vector decomposition is largely preserved in central vestibulo-ocular reflex (VOR) pathways to directly couple specific canals to specific ocular-motor outputs (Cohen et al. 1964; Davidovics et al. 2013; Suzuki and Cohen 1964; Suzuki et al. 1964, 1969). Although both regular and irregular discharging afferents contribute to the VOR, units with regular discharge statistics dominate inputs driving the low-frequency VOR (Lasker et al. 1999; Minor and Goldberg 1991; Minor and Lasker 2009). To good approximation, the action potential firing rate of these regularly discharging units encode displacement of the cupula (e.g., Fig. 3*F*) (Fernandez and Goldberg 1971). In contrast, other units encode rapid transient changes in cupula displacement, exhibit excitatory-inhibitory nonlinearity, and exhibit adaptive run down for a maintained cupula displacement (e.g., Fig. 3*G*), analogous to responses of irregularly discharging units in mammals (Lasker et al. 2008; Ramachandran and Lisberger 2006; Sadeghi et al. 2007a). These phasic units also contribute to the VOR and are preferentially activated during rapid angular movements in the excitatory direction of the canal. This preferential directional sensitivity is why head-impulse tests, which apply small rapid rotations in the excitatory direction of a canal, can be used clinically to selectively excite and evaluate individual semicircular canals (Halmagyi et al. 2017; McGarvie et al. 2015).

All hair cells are oriented in the same direction in the crista and are putatively activated together by deflection of hair bundle kinocilia tethered to the cupula. Anterior canal (AC) and posterior canal (PC) hair cells are oriented to be excited for ampullofugal endolymph displacement (toward the slender duct) while lateral canal (LC) hair cells are oriented to be excited by ampullopetal endolymph displacement. Individual vestibular afferent neurons can receive quantal synaptic inputs from type II hair cells and both quantal and nonquantal synaptic inputs from type I hair cells (Contini et al. 2012; Holt et al. 2007; Holt et al. 2006; Lim et al. 2011; Sadeghi et al. 2014; Starr and Sewell 1991; Yamashita and Ohmori 1990). Although the nonquantal component has a rapid onset and likely contributes to high-frequency sensitivity (Eatock 2018; Eatock and Songer 2011; Songer and Eatock 2013), it is the quantal component that exhibits substantial adaptive run down over time consistent with adaptive properties of and phase locking of irregularly discharging afferent neurons (Furukawa et al. 1978; Furukawa and Matsuura 1978; Goutman 2012; Highstein et al. 2014, 2015; Li et al. 2014). Inputs from multiple hair cells drive individual afferent neurons, which generate action potentials primarily through voltage-sensitive ion channels and the interaction of nonlinear voltage-sensitive and calcium-sensitive ion channels, a dynamic that further shapes the temporal code (Eatock 2018; Goldberg and Holt 2013; Kalluri et al. 2010; Li et al. 2010; Meredith and Rennie 2016; 2015; Songer and Eatock 2013). Signal processing by hair cells and afferent neurons is controlled by the CNS through the action of efferent synaptic contacts in the crista (Goldberg et al. 2012; Holt et al. 2017; Lee et al. 2017; Poppi et al. 2018). These factors determine how the neural code represents the time-dependent motion of sensory hair bundles, with each individual afferent neuron preferentially encoding specific temporal features of the stimulus and modulating sensitivity with stimulus direction. As discussed in subsequent sections, disorders altering canal mechanics can impact coding by certain afferent neurons more than others primarily because of phasic versus tonic sensitivity imparted the biophysics of hair-cell/afferent complexes.

### Macromechanics

As summarized above, each labyrinth includes three endolymph filled membranous tubes (Fig. 1, *A* and *B*) immersed in three rigid perilymph-filled annular tubes (Fig. 1*D*). To gain insight into the fluid mechanics of the semicircular canals, it is useful to estimate the ratio of steady inertia to viscous drag quantified by the Reynolds number *R*_e_ = ρ*aV*/μ, and the ratio of unsteady inertia to viscous drag quantified by the Womersley number $W_{o}=\sqrt{\rho \omega a^{2}/\mu }$. In these expressions ρ (~1,000 kg/m^3^) is the density, μ (~9 × 10^−4^ Pa/s) is absolute fluid viscosity, *a* is the characteristic radius of the canal duct, *V* is the characteristic velocity of endolymph relative to the labyrinth wall, and ω = 2π*F* (rad/s), where *F* (Hz) is the frequency of the stimulus (e.g., sinusoidal angular head oscillation). In humans, the characteristic frequency of volitional head movements is on the order of *F* = 1 Hz, and for air conducted sound or bone conducted vibration the characteristic frequency is on the order *F* = 1,000 Hz. The characteristic radius of the human ampulla is ~5 × 10^−4^ m, and the characteristic radius of the slender duct is ~1.5 × 10^−4^ m (Ifediba et al. 2007). Based on direct measurements in toadfish, which has dimensions similar to humans, the fluid velocity in the ampulla is estimated to be on the order *V* = 1 × 10^−6^ m/s (Fig. 3, *D* and *E*) and in the slender duct is estimated to be on the order *V* = 1 × 10^−5^ m/s in adult humans (Rabbitt et al. 2009, 2010). With the use of these orders of magnitude, the Reynolds number is on the order 10^−3^ for volitional head movements. This means the convective nonlinearity in the Navier-Stokes equations is small and, to very good approximation, the fluid motion does not involve turbulence. With the use of the same values, the Womersley number *W*_o_ is on the order of 1. Unsteady inertia begins to have an important impact on viscous drag and fluid motion if *W*_o_ > 2π, so unsteady effects are also unimportant in the canals for common volitional head movements. Hence, the fluid mechanics is governed by viscous Stokes flow for most volitional head movements, and relatively simple ideas and models can be used to understand canal fluid mechanics for low- to mid-frequency volitional or imposed head movements.

Shortly after J. Goldberg and C. Fernandez began their examination of semicircular canal afferent responses to angular motion stimuli, C. Oman, L. Young, and others began to examine the contribution of canal biomechanics and morphology to directional and temporal coding (Curthoys and Oman 1987; Oman et al. 1979, 1987; Selva et al. 2009). Expanding on the early torsion-pendulum model of Steinhausen (1933), a simple rigid-labyrinth model has emerged that captures the major features of canal macromechanics for movements in the low- to mid-frequency range (<6 Hz in humans) (Ifediba et al. 2007; Oman et al. 1987; Rabbitt et al. 2003). For a single canal, this “rigid” duct model takes the form of a simple differential equation describing cupula volume displacement *Q*(*t*) evoked by angular head movements:

(1) $$m\frac{d^{2}Q}{dt^{2}}+c\frac{dQ}{dt}+kQ=f.$$

The parameters *m*, *c*, *k*, and *f* are completely determined by the three-dimensional morphology, physical properties of the endolymph, stiffness of the cupula, and angular motion direction and time course. *Equation 1* can be derived from first principles of physics using one-dimensional conservation of mass and momentum applied to the endolymphatic loop (Oman et al. 1987; Rabbitt et al. 2003) or using an asymptotic expansion of the 3D Navier-Stokes equations reduced for incompressible Stokes flow (Damiano 1999; Rabbitt and Damiano 1992). In *Eq. 1*, *k* is the volumetric stiffness of the cupula, *c* is the hydraulic resistance of the slender duct, *m* is the hydraulic mass of the slender duct, and *f* is the inertial pressure force generated by accelerating the fluid loop. The hydraulic mass *m* can be set to 0 for movements in the low- to mid-frequency range under most conditions (<6 Hz in humans). We should stress that earlier reports include the hydraulic mass *m* and simulate responses above 6 Hz (Oman et al. 1987; Rabbitt et al. 2003), but high-frequency predictions using *Eq. 1* are suspect because membranous labyrinth deformation is not included (Grieser et al. 2016; Iversen and Rabbitt 2017; Iversen et al. 2018).

Precise expressions to determine parameters in *Eq. 1* from the morphology and physical properties are available (Oman et al. 1987; Rabbitt et al. 2003), but these parameters can also be approximated using very simple estimates. For low- to mid-frequency movements, the cupula volumetric stiffness can be approximated using $k=\gamma h\lambda /A_{c}^{2}$, where γ is the elastic shear modulus of the cupula, *A*_c_ is its frontal area of the cupula, and *h* is the thickness of the cupula. The hydraulic resistance can be approximated using $c=\mu l\lambda /A^{2}$, where μ is the endolymph viscosity, l is the length along the centerline of the curved slender duct, and *A* is the cross-sectional area of the slender duct. The parameter λ ≈ 8π for low-frequency movements (<6 Hz) is based on the Hagen-Poiseuille paraboloid velocity profile in a circular duct (Iversen et al. 2017). The hydraulic resistance “*c*” is identical to the hydraulic resistance commonly used in analysis of low Reynolds number tube flow. For angular rotations of the head, the inertial forcing has units of pressure and arises from acceleration of the loop of endolymph. It can be approximated using

(2) $$f=f_{acc}=\left[2\pi \rho R^{2}\cos(\beta_{m})\right]\ddot{\Omega },$$

where ρ is the endolymph density, *R* is the characteristic radius of the canal loop, β_m_ is the angle between the maximum response direction and the angular rotation direction, and $\ddot{\Omega }$ (rad/s^2^) is the angular acceleration of the head about the rotation axis. It’s important to note for a circular canal this driving pressure does not depend on canal cross-sectional area of the duct.

The rigid canal model predicts that, following a transient head movement, the cupula will return to its original undeformed position following an exponential relaxation [$Q(t)=Q_{0}e^{-t/\tau }$] with mechanical time constant approximated by

(3) $$\tau =\frac{c}{k}=\frac{\mu lA_{c}^{2}}{\gamma hA^{2}}.$$

**Fig. 4.**
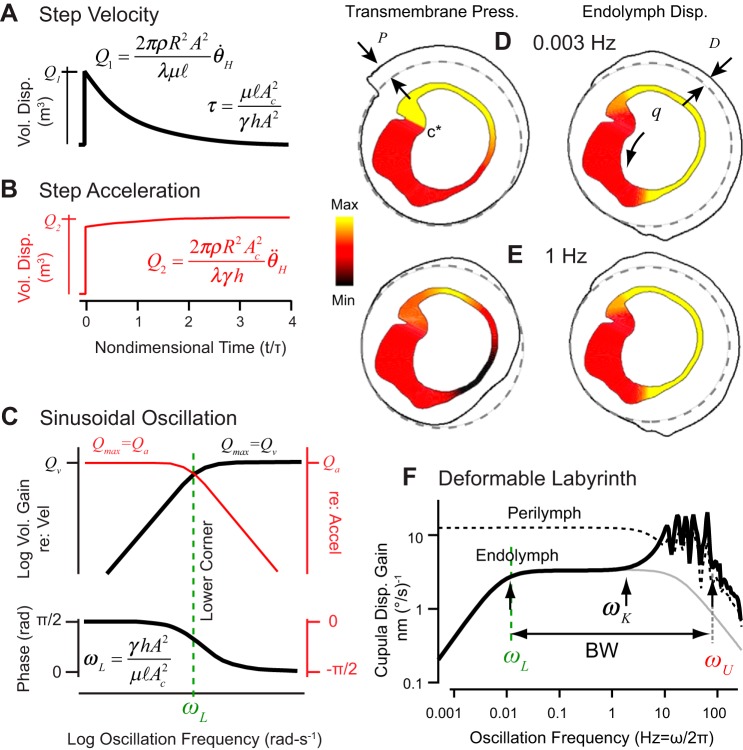
Semicircular canal macromechanics. *A* and *B*: rigid labyrinth model prediction for transient cupula volume displacement in response to a step excitatory increase in angular velocity (c.f. Fig. 3*C*) and for sustained cupula volume displacement in response to a step angular acceleration. *C*: bode plots showing cupula volume displacement gain and phase as a function of frequency for sinusoidal oscillation of the head. Left axes show magnitude and phase relative to angular head velocity (flat gain above ω_L_), while right axes are relative to angular head acceleration (flat gain below ω_L_). *D* and *E*: pressure and endolymph displacement as a function of position in the horizontal canal membranous duct for sinusoidal rotation at 0.003 Hz (*D*) and at 1 Hz (*E*) (yellow: max; red: zero; and black: min). *F*: membranous duct deformability alters the high-frequency cupula displacement (thick black) relative to a perfectly rigid canal (gray). Deformability increases the bandwidth (BW) by shifting ω_U_ up and increases high-frequency sensitivity through the role of perilymph deforming the canal. [*D*–*F*: adapted from Iversen and Rabbitt (2017).]

The relaxation time constant τ is the ratio of hydraulic resistance (*c* arising from the slender duct) to volumetric stiffness (*k* arising from the cupula) and has been measured experimentally in toadfish (Fig. 3, *A* and *E*) (Rabbitt et al. 2009, 2010). The cupula relaxation time constant in humans is estimated to average ~16–20 s (corresponding to a lower corner ω_L_= 1/τ = 0.055 rad/s ~0.01 Hz) but is likely to vary considerably between individuals due primarily to variability in viscoelastic properties of the cupula extracellular matrix associated with age or other factors. For a step increase in angular velocity of the head, the inertial forcing on the right hand side of *Eq. 1* is an impulse Dirac delta function. The cupula volume displacement in this case makes a rapid jump, followed by an exponential relaxation back to zero, consistent with direct experimental observation shown in Fig. 3, *C* and *E*, and relaxation simulated by *Eq. 1* (shown in Fig. 4*A*). Concomitantly, a step increase in angular acceleration evokes a maintained cupula volume displacement as simulated and shown in Fig. 4*B*.

Since *Eq. 1* is linear, sinusoidal head oscillations result in sinusoidal cupula volume displacement with a specific gain and phase relative to the stimulus (e.g., Fig. 3*D*). Although nonlinearity has been observed in semicircular canal cupula motion in vivo (Rabbitt et al. 2010), it is very small relative to the linear component and can be neglected under almost all conditions. For sinusoidal oscillation of the head the angular acceleration is $\ddot{\Omega }={\ddot{\theta }}_{H}e^{j\omega t}$, where ${\ddot{\theta }}_{H}$ (rad/s^2^) is the magnitude of angular head acceleration ω (rad/s) is the oscillation frequency. For frequencies below the lower corner frequency (ω < ω_L_) the canal acts as a broadband angular acceleration detector (detecting ${\ddot{\theta }}_{H}$ with flat gain and zero phase), and above the lower corner frequency (ω < ω_L_), the canal acts as a broadband angular velocity detector (detecting ${\dot{\theta }}_{H}$ with flat gain and zero phase). This is illustrated in Fig. 4*C*, where the sinusoidal cupula volume displacement is plotted in Bode form of gain and phase as a function of oscillation frequency, relative to angular velocity (black) and relative to angular acceleration (red). The volume displacement in these two frequency ranges can be estimated by

(4) $$Q=\{\begin{array}{ccc} Q_{a}=\left(2\pi \rho R^{2}A_{c}^{2}/\lambda \gamma h\right){\ddot{\theta }}_{H}, & \omega <\omega_{L} & \left(a\right)\\ Q_{v}=\left(2\pi \rho R^{2}A^{2}/\lambda \mu l\right){\dot{\theta }}_{H}, & \omega >\omega_{L} & \left(b\right) \end{array}.$$

Below ω_L_ the response is analogous to a simple spring scale, where the inertial force from acceleration the endolymphatic loop is balanced by elastic deflection of the cupula. Above ω_L_ the response is analogous to pressure driven viscous flow in a tube, where the inertial force from acceleration of the endolymphatic loop is balanced by viscous drag in the long and slender duct. Stiffness of the cupula is not important above ω_L_, and viscosity of the endolymph is not important below ω_L_.

The “rigid” model has been extended to the full three-dimensional labyrinth by coupling the three membranous canals together at bifurcation points (David et al. 2016; Ifediba et al. 2007; Rabbitt 1999). In this case, *Eq. 1* becomes a vector equation for volume displacements in all three canals. This 3D approach is useful to examine how the morphology of the labyrinth underlies directional coding, and to evaluate canalith repositioning procedures (described in *Benign Paroxysmal Positional Vertigo*). Fluid displacement, and hence canal sensitivity, are maximized for angular rotations about an axis nearly perpendicular to the plane of the canal. Anatomical canal planes measured in humans are illustrated in Fig. 1*E* (Della Santina et al. 2005). Double arrow vectors indicate directions perpendicular to anatomical plane that maximally excite the left AC and right PC, right AC and left PC, left LC, and right LC. These anatomical directions correspond closely to prediction of the maximum response directions based on fluid mechanics in a 3D rigid canal model of the human membranous labyrinth, shown in Fig. 1*F* for the left LC (Ifediba et al. 2007). Directional sensitivity (<6 Hz) follows the 3D cosine rule, where there is no response for rotations within the null plane of the canal and maximum response for rotations perpendicular to the null plane. Each canal nerve therefore encodes one vector component of a 3D head movement, which can be estimated by morphology of the canals (Blanks et al. 1985, 1975; Blanks and Torigoe 1989; Calabrese and Hullar 2006; Della Santina et al. 2005; Hullar et al. 2005; Ifediba et al. 2007; Sadeghi et al. 2009). Vector decomposition by the cosine rule has been shown to apply even in animals that have more complex nonplanar canal morphology (Dickman 1996), but determining the direction from the morphology is more complex (Ifediba et al. 2007). The maximum response directions from the three canals are nearly orthogonal to each other in all species studied to date, even if the canals are complex in morphology. When the maximum response directions are nearly orthogonal, rotation about the maximum response direction of a single canal evokes almost no mechanical responses in the sister canals. Orthogonality simplifies neural computations and convergence in the CNS required for the 3D VOR and other motor outputs, putatively an advantage for the animal. Responses of the sister canals can be completely eliminated if the rotation is in the null plane of both sister canals. This special direction is called the “prime” direction and defines the axis of rotation that excites only one canal (Ifediba et al. 2007; Rabbitt 1999). Prime directions define the mathematical eigenvectors along which 3D angular head movements are transmitted to the CNS by the three canal nerve branches. From a practical point of view, the prime directions and maximum response directions are close to each other and can be approximated by vectors perpendicular to each anatomical canal plane (Ifediba et al. 2007).

The rigid model can be extended to include unsteady inertia by making λ frequency dependent (Iversen and Rabbitt 2017; Rabbitt et al. 2003), but even with this addition the rigid model fails at high frequencies primarily because membranous duct deformability is not included. The simple rigid model in *Eq. 1* does not include spatial distribution of cupula strain, hair bundle displacements across the sensory epithelium, or fine multidimensional fluid displacements in large regions of the labyrinth such as the utricle and ampullae. Computational methods have been applied to capture some of these macromechanical complexities (David et al. 2016; Iversen and Rabbitt 2017; Selva et al. 2009, 2010; Shen et al. 2013; Wu et al. 2011; Yamauchi et al. 2002), with membranous duct deformability being the most relevant to pathological responses and clinical presentations described in later sections.

## MICRO- AND NANOMECHANICS

Although semicircular canal macromechanics is responsible for directional coding and aspects of temporal coding and sensitivity, macromechanics cannot describe diversity in afferent responses or differences in temporal coding at the level of individual afferent neurons (Fig. 3). Part of the difference between macromechancis and neural coding arises from micromechanics and nanomechancial gating of hair cell MET currents. An extensive body of work by Hudspeth and colleagues has identified several fundamental aspects of mechanotransduction in hair cells. MET currents can exhibit extensive adaptation in both auditory and some vestibular hair cells, contributing significantly to the temporal code carried by afferent neurons (Eatock et al. 1987; Howard and Hudspeth 1987; Ricci et al. 2005; Songer and Eatock 2013; Wu et al. 1999). Data primarily from hair cells with short bundles in a variety of organs and species demonstrate that both micro- and nanomechanical factors contribute to MET current kinetics. An unconventional myosin motor protein in the hair bundle putatively mediates tip link tension, contributes to adaptation of MET gating, and sets the operating point of MET channels (Gillespie and Cyr 2004). On a faster time scale, the calcium component of the MET current is involved in active force generation and setting the resting state of hair cells (Fettiplace and Kim 2014). Micromechanics of the lipid bilayer at the tips of stereocilia is likely involved in nanomechanical gating and kinetics of MET channels (Gianoli et al. 2017; Kim 2015; Peng et al. 2016; Powers et al. 2012). Micromechanics of the bundle itself also plays an important in the relationship between gross bundle motion and MET channel gating. There is evidence from auditory hair cells that bundles might change length during transduction (Hakizimana et al. 2012), a phenomena that could contribute to MET current kinetics and potentially to active bundle movements (Breneman et al. 2009). Furthermore, hair bundle micromechanics might lead to splay between individual stereocilia under some physiological conditions (Nam et al. 2015) but potentially not in all hair cell types or organs (Flock et al. 1977; Kozlov et al. 2007). These biophysical and biomechanical factors have been identified and studied primarily in auditory and vestibular hair cells with short bundles, and their relative importance is not yet clear in mature semicircular canal hair cells with very long bundles. Semicircular canal hair cells in mice and other rodents are short at birth and require ~2 wk of postnatal development to reach adult length. Hence, data from developing animals would not be expected to reflect the micromechanics of the mature organ, potentially contributing to maturation of coding. Micro- and nanomechanical data from mature semicircular canal hair cells are sparse, in part due to the technical challenge of experiments with long and delicate semicircular canal hair bundles.

## SEMICIRCULAR CANAL MECHANICS AND ANIMAL BEHAVIOR

The importance of labyrinth morphology and macromechanics for angular sensitivity and directional coding has led to the hypothesis that morphological specializations might reflect movement lifestyle of the animal, specializations putatively appearing through natural selection driven by sensory coding objectives. If true, analysis of labyrinth morphology might lend insight into the possible behavior of extinct animals, e.g., Araujo et al. (2018); Jones and Spells (1963); Muller (1999); Spoor et al. (2007); Spoor and Zonneveld (1998). Based on fossil records it is often possible to estimate the planar orientations of the bony canals within the skull, providing a good indicator of maximum response directions (Ifediba et al. 2007), ocular muscle pulling directions, and directional organization in the CNS (Suzuki et al. 1964; Suzuki et al. 1969). The orientation of the LC is perhaps the most relevant to imply the direction of volitional head rotations due to its relationship to horizontal compensatory eye movements established in extant species.

Implications beyond directional coding are difficult to make based on bony labyrinth morphology alone. The radius *R* and length l of each canal are two key geometric factors contributing to mechanical sensitivity (*Eq. 4*) that can be estimated from the bony labyrinth, but the membranous duct cross sectional area *A*, the cupula frontal area *A*_c_, and cupula thickness *h* and elastic properties are equally important but cannot be directly determined from the bony labyrinth. If all dimensions of the membranous labyrinth scaled between species in proportion to bony labyrinth size, the relaxation time constant (or ω_L_ = 1/τ) (*Eq. 3*) would be independent of canal size, while sensitivity would increase with canal size (*Eq. 4*) (Highstein et al. 2005). Although there is evidence of reduced mechanical sensitivity in small canals (Lambert et al. 2008), scaling all dimensions together cannot account for diversity of afferent sensitivity to angular motion in extant species (Hullar 2006). For example, relative to mammals the cross-sectional area of the membranous duct (*A*) in turtles is very large given the canal size (*R*), which extends the bandwidth and sensitivity to angular acceleration (lower τ, *Eq. 3*) at the expense of angular velocity bandwidth. This favors detection of slow movements putatively important to the turtle, but comes at the expense of angular velocity sensitive bandwidth (*Eq. 4*), which would be important to the VOR in mammals to accommodate rapid head movements. This distinction cannot be obtained directly from morphology of the bony labyrinth. Further complicating interspecies comparisons are differences in signal processing by hair-cell/afferent complexes. Some canal afferent neurons modulate discharge rate in proportion to deflection of the cupula, while others show varying degrees of adaptation (e.g., Fig. 3). The adaptation resembles a fractional derivative (Holstein et al. 2004) and appears as frequency-dependent gain and phase for sinusoidal movements. The extent of postmechanical signal processing varies between species and afferent neuron types, with irregularly discharging calyx-bearing afferents exhibiting the most adaptation in mammals (Baird et al. 1988; Blanks and Precht 1976; Boyle and Highstein 1990; Fernandez and Goldberg 1971; Goldberg et al. 2012; Goldberg and Fernandez 1971b; Honrubia et al. 1989; Hullar et al. 2005; Kim et al. 2011; Lambert et al. 2008; Paulin and Hoffman 1999; Songer and Eatock 2013). Given these factors, estimating directional coding from bony labyrinth morphology is likely reliable, but estimating properties of temporal coding requires some additional information.

## MECHANICS OF THE SEMICIRCULAR CANALS IN DISEASE

### Damaged or Regenerating Cupula

**Fig. 5.**
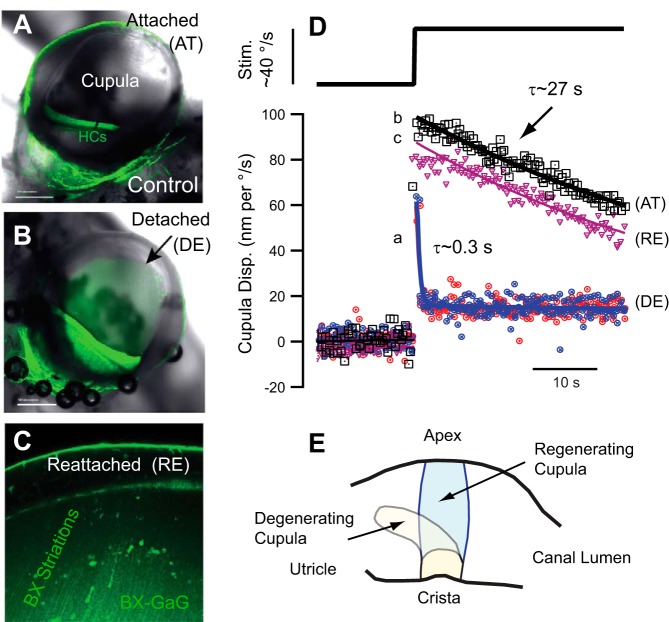
Detachment and regeneration of the cupula. *A* and *B*: the normally transparent cupula is attached around its entire periphery in the control condition [hair cells (HCs); *A*] but can become detached at the apex if excessive transcupular pressure is applied (*B*). *C*: fluorescent xyloside (BX, green) introduced into the endolymph enters the Golgi apparatus of cells and reveals increased production of GaGs in the sensory epithelium that migrate up the cupula toward the detached apex, putatively reflecting a process of cupula self-repair (toadfish model). *D*: the detached cupula (DE) responds with an extremely rapid relaxation time constant relative to the predamage condition (AT) and relative to the reattached condition (RE). Reattachment can occur in ~7 h if the detachment is modest and confined to the apex. *E*: experimental evidence suggests a severely damaged cupula can be replaced by a regenerated cupula, at least in fish. [*D* and *E*: adapted from Rabbitt et al. (2009).]

As illustrated in Fig. 2, semicircular canal hair cell kinocilium is tethered deep into the cupula resulting in sensory hair bundle displacements that putatively track cupular displacement. This relationship can be lost if the cupula is damaged. Mechanical trauma, including rapid compression of the membranous duct, has been shown in animal models to detach the cupula from the ampulla at its apex, a location where it normally adheres to epithelial cells lining the inside surface of the ampulla (Fig. 5*B*) (Hillman 1974; McLaren and Hillman 1979; Rabbitt et al. 1999, 2009). Fluorescence in Fig. 5, *A*–*C*, green, is from a xyloside (BX) administered to the endolymph in the toadfish model, which allows visualization of the epithelium and the cupula in vivo (Holman et al. 2016). Cupula detachment results in dramatically reduced sensitivity of the canals to angular motion stimuli because endolymph flows over the top of the cupula instead of deflecting it (Rabbitt et al. 2009). Relatively weak adhesion of the cupula to the ampulla at the apex acts as a pressure relief valve offering mechanical protection. Detachment and pressure relief putatively protect hair cells from overstimulation at the expense of temporary dysfunction. A healthy attached cupula responds to a step angular velocity stimulus with an immediate displacement followed by a slow relaxation back to its resting position (Figs. 3*C* and 5*E*). After detachment, the cupula has very little response to the same step stimulus, showing only a quick jerk at the onset of the stimulus followed by a maintained offset that does not completely relax to the prestimulus position (Fig. 5*E*). This mechanical damage virtually eliminates angular motion sensitivity even if the sensory hair cells and afferent neurons are perfectly capable of responding.

For modest traumatic detachment at its apex, the cupula can reattach as soon as 7 h after damage, at least in the toadfish model (Fig. 5, *C* and *E*). Administration of BX into the endolymph provides some insight into the self-repair process (Holman et al. 2016). Following administration, BX enters the Golgi apparatus of hair cells and supporting cells in the sensory epithelium, where it primes the production of fluorescently labeled GaGs. Extracellular BX fluorescence develops in the cupula over time, as GaGs primed in the epithelium migrate into the cupula toward the site of detachment at the apex. Fluorescent striations running from the sensory epithelium toward the apex develop, with reattachment of the cupula and recovery of canal function occurring over a similar time course. Although BX-primed GaGs are not present in untreated control tissue, their migration would be expected to indicate the time course of endogenous GaG migration in the cupula, thus suggesting a slow process of self-maintenance and repair is likely ongoing (Holman et al. 2016).

In one unusual experimental case, we observed two cupulae in a single ampulla of the toadfish model in vivo. One cupula was deflected toward the utriculus and was optically milky, while the second cupula was located in the normal position and was optically transparent (Rabbitt et al. 2009). We hypothesized that the deflected cupula had become detached beyond the capacity for self-repair and the replacement cupula putatively regenerated from the epithelium extending over time to the apex of the ampulla. Single unit afferent recordings from this particular animal in response to angular motion stimuli revealed unusual properties with overall reduced sensitivity, increasing gain with frequency, and advanced phase relative to controls. These changes in sensitivity are consistent with a mechanical origin if the new cupula was leaky and allowed some endolymph to pass through, or over the apex, during this stage of regeneration (Damiano 1999; Rabbitt et al. 2003). Observations suggest the cupula has the capacity for complete regeneration, presumably requiring many days longer than simple reattachment at the apex.

### Light or Heavy Cupula

**Fig. 6.**
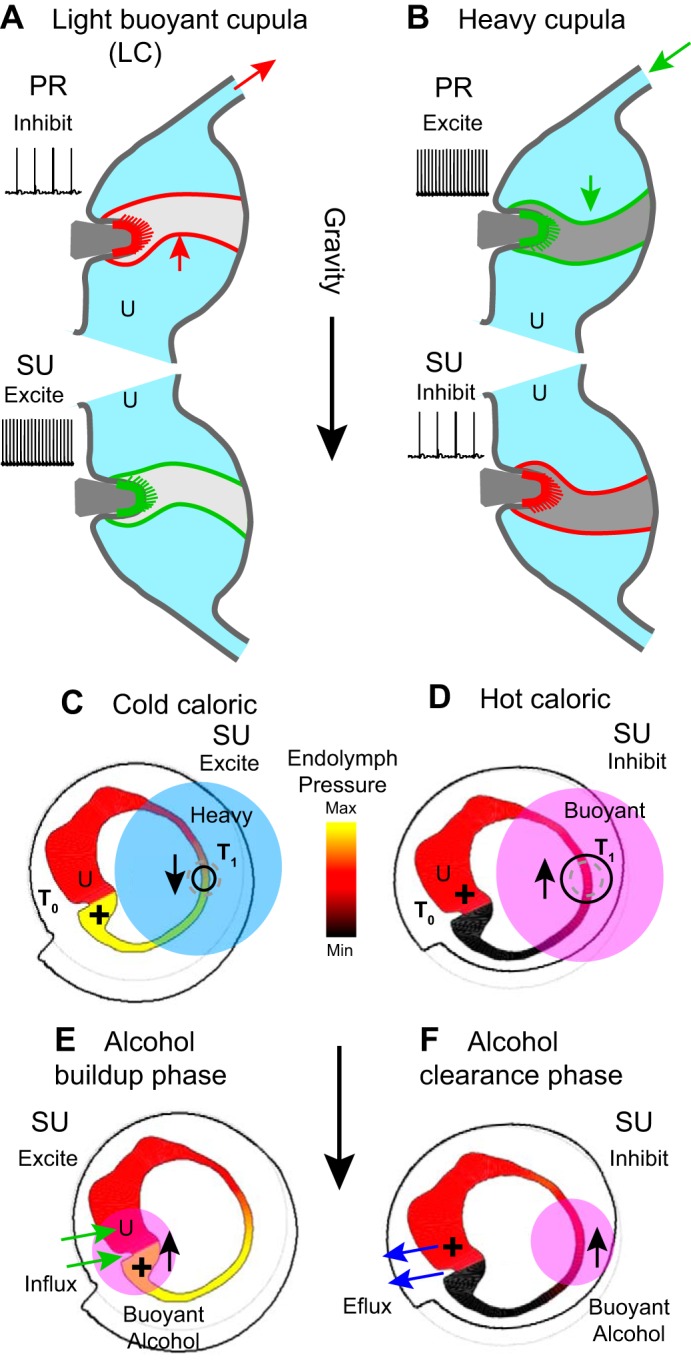
Light cupula, heavy cupula, caloric, and alcohol buoyancy. *A*: under pathological conditions the specific gravity of the cupula can be abnormally low (light cupula) causing it to be buoyant and deflect up when oriented relative to gravity. Light cupula evokes a sustained decrease in lateral canal (LC) afferent nerve discharge rate in the prone (PR) position and a sustained increase in discharge rate when in the supine (SU) position. *B*: heavy cupula evokes the opposite neural responses. *C*: cooling the long slender limb of the lateral canal by cold irrigation of the ear canal increases the specific gravity of a section of endolymph, thus generating a sustained pressure (+) across the cupula and sustained afferent nerve responses similar to light or heavy cupula. *D*: hot caloric irrigation evokes the opposite mechanical responses. *E*: diffusion of alcohol into the endolymph from the blood can also generate gravity-dependent responses through buoyancy. Influx of alcohol from capillaries in the end organs causes a buildup of alcohol concentration lowering the specific gravity of the cupula and endolymph near the organs. This renders the canals sensitive to gravity, very similar to light cupula. *F*: during alcohol clearance, the endolymph density near the sensory organs temporarily decreases relative to the slender duct, thus reversing the direction of the net buoyancy force.

Under normal physiological conditions the specific gravity of the cupula matches the endolymph, so tilting the head relative to gravity does not generate any buoyancy force in the cupula or gravity-dependent neural responses. This match can change under some pathological conditions. There is evidence in patients presenting with direction-changing positional nystagmus that a pathologically “heavy” or “light” cupula induces semicircular canal sensitivity to gravity, geotropic, or antigeotropic, respectively (Hiruma et al. 2011; Kim et al. 2014; Schubert et al. 2017; Shin et al. 2017; Tomanovic and Bergenius 2014). Figure 6*A* illustrates sustained cupula deflections and neural responses occurring when the LC is positioned relative to gravity to deflect a “light cupula” by buoyancy force. The LC response is inhibitory when positioned in the prone position and excitatory when positioned in the supine position. Although the precise etiology is unknown, “light cupula” could occur due to a condition of the endolymph causing abnormally high density or a condition of the cupula causing abnormally low density. Transient decreases in cupula density could potentially occur following damage due to low concentrations of heavy macromolecules during regeneration. It is equally possible that increases in endolymph density could potentially occur due to any number of inner ear or systemic conditions altering endolymph macromolecular and/or small molecule composition. Given these potential molecular sources of light cupula, one might expect the condition to resolve in some cases with systemic conditions, without targeted treatment. Heavy cupula, illustrated in Fig. 6*B*, presents with opposite polarity relative to gravity. Although it could arise from conditions impacting the cupula and/or endolymph, in most cases it is thought to occur when dense calcium carbonate-rich particles become adhered to the cupula, a condition termed cupulolithiasis (Buki et al. 2014; Schuknecht and Ruby 1973; Shin et al. 2017). Cupulolithiasis is one cause of heavy cupula, but heavy cupula could arise from other conditions including high macromolecular content or calcium precipitation within the cupula itself. Cupulolithiasis generates maintained gravity-dependent forces acting on the cupula and sustained gravity dependent nystagmus.

A light or heavy cupula causes nystagmus because the cupula floats or sinks in the endolymph. *Equation 1* can be used to estimate the cupula volume displacement caused by a mismatch between cupula density ρ_c_ and endolymph density ρ. The mismatch generates a pressure forcing term in *Eq. 1*

(5) $$f=f_{cup}=hg\left(\rho -\rho_{c}\right)\cos(\beta_{c}),$$

where *g* is the acceleration of gravity and β_c_ is the angle between the direction of gravity and a vector perpendicular to the cupula. Upon reorientation of the head (changing β_c_), the cupula will displace following the relaxation time constant (*Eq. 2*) to reach a steady-state volume displacement

(6) $$Q_{c}=\frac{g\left(\rho -\rho_{c}\right)A_{c}^{2}}{8\pi \gamma }.$$

Notice the thickness of the cupula does not appear in this final expression because it contributes to both the driving buoyancy force *f* and in the restoring hydraulic stiffness *k* .

### Caloric Stimulation

Introduction of caloric irrigation at the turn of the 20th century by Robert Bárány provided a means to deliver long-lasting unilateral stimuli allowing clinical examination of canal function and central vestibular pathways (Bárány 1906, 1916; Fitzgerald and Hallpike 1942). Irrigation of the external ear canal by cold or hot water (or other media) enables gravity-sensitive responses, with slow phase eye movements changing direction with orientation of the head relative to gravity. Caloric stimulation was originally thought to activate the lateral semicircular canal by thermal buoyancy induced “convective endolymph flow,” but now we know there is no true convective flow. It has since been established that thermal buoyancy of the endolymph is the dominant mechanism (Valli et al. 2002–2003) but acts to generate sustained pressure on the cupula in the absence of a true convective flow (Gentine et al. 1990). Caloric irrigation also has a gravity-independent component, most clearly demonstrated by vestibular responses during space flight and microgravity (Clarke et al. 1988; Oosterveld et al. 1991; Scherer et al. 1986). The gravity-independent component has both mechanical (Gentine et al. 1991; Kassemi et al. 2005a, 2005b; Pau and Limberg 1990) and neurophysiological substrates (Klinke 1992; Park et al. 2010; Rabbitt et al. 2016; Rajguru et al. 2011; Young and Lowry 1994). In total, caloric irrigation evokes semicircular canal responses consisting of three distinct components: *1*) gravity-dependent thermal buoyancy, *2*) gravity-independent thermal expansion, and *3*) gravity-independent temperature-dependent hair cell/neuron excitability. The contribution of buoyancy and thermal expansion can be quantified on the basis of first principles of mechanics.

Thermal buoyancy arises from the temperature gradient across the labyrinth (Fig. 6, *C* and *D*, T_1_ vs. T_0_), which generates a concomitant density gradient in the endolymph and gravity-sensitive pressure across the cupula. The magnitude and time course of the cupula response can be analyzed from first principles using computational models of heat conduction through the 3D temporal bone and fluid mechanics in the labyrinth (Kassemi et al. 2005b; Shen et al. 2013) or can be approximated using the simplified model introduced above by replacing the angular acceleration forcing in *Eq. 1* with a thermal buoyancy derived term. The net pressure on the cupula caused by buoyancy is found by integrating the gravity-dependent weight of the endolymph tangent to the canal centerline around the canal loop. For the case when the gravitational vector is in the canal plane, the buoyancy force *f* in *Eq. 1* is

(7) $$f=f_{b}=\pi \rho R^{2}\alpha (T_{1}-T_{0})g,$$

where α ~ 0.0003 (1/°C) is the thermal expansion coefficient of endolymph. This buoyancy pressure is present only under the action of gravity (*g* = 9.8 m/s^2^) and requires a thermal gradient across the canal loop (Fig. 6, *C* and *D*, *T*_0_ ≠ *T*_1_). For a prescribed orientation of the head and a slowly changing thermal gradient, the buoyancy force is constant and leads to a steady-state cupula volume displacement of approximately

(8) $$Q_{b}=\frac{\pi \rho R^{2}A_{c}^{2}}{\lambda \gamma h}\alpha (T_{1}-T_{0})g$$

If the temperature is changed rapidly, the tonic buoyancy force (*Eq. 7*) must be augmented by a transient arising from thermal expansion. The direct expansion effect is similar to mechanical indentation of the membranous duct, which has been shown to evoke transient displacements of the cupula that relax over time following the time constant in *Eq. 3* (Dickman and Correia 1989; Rabbitt et al. 1995, 2009, 2010). Imposing a temperature gradient across the loop is roughly equivalent to injecting a volume of $dV\approx lA\alpha (T_{1}-T_{0})$ into the endolymph of the slender duct. Based on Rabbitt et al. (1995), this drives cupula volume displacement in *Eq. 1* through a gravity-independent pressure force

(9) $$f_{e}=\zeta \rho lA\alpha \frac{d(T_{1}-T_{0})}{dt}$$

where ζ is a scaling factor relating volumetric injection to equivalent head velocity (ζ ~200 rad·s^−1^·mm^−3^). This driving pressure is proportional to the time rate of change of the temperature gradient as opposed to the temperature gradient itself. As a result, thermal expansion generates a transient cupula displacement with kinetics constrained by *Eq. 1*. In the LC, the transient is inhibitory for cold caloric irrigation (Fig. 6*C*, indicated by small black contracting circle) and excitatory for hot caloric irrigation (Fig. 6*D*, indicated by black expanding circle). Hence, thermal expansion adds a transient response in the same direction as buoyancy in the prone position but in the opposite direction in the supine position. The expansion term is relevant to responses in zero gravity and in the buoyancy null plane, but the effect is small relative to the buoyancy term in the maximum gravity-sensitive position and dissipates for slow caloric irrigations lasting >30 s (*d*/*dt* approaches 0). Both thermal buoyancy (*Eq. 7*) and expansion (*Eq. 8*) act on all three canals but differ in magnitude primarily because the temperature gradient generated by external ear canal irrigation is much larger across the LC relative to the sister canals.

The two mechanical effects of caloric irrigation quantified above are augmented by the direct action of temperature on biophysics of ion-channels and proteins responsible for temperature-dependent changes in synaptic transmission and afferent neuron discharge rate (Klinke 1992; Park et al. 2010; Rabbitt et al. 2016; Rajguru et al. 2011; Young and Lowry 1994). In practice, the orientation of the head that nulls the caloric response for long-duration irrigations (>30 s) zeros the combined direct biophysical effect and the mechanical buoyancy effect. Inhibition by cold irrigation on the neurophysiological level is countered in the caloric null orientation by a small buoyancy-driven excitation resulting in minimal eye movements.

### Alcohol Positional Nystagmus

Money et al. (1965) provided the first direct evidence that alcohol positional nystagmus arises from action on the semicircular canals. It was subsequently shown that nystagmus followed a two-component time course with the first phase consistent with conditions of a light cupula and the second phase consistent with conditions of a heavy cupula (Koizuka et al. 1989). Administration of heavy water instead of alcohol reversed direction of the nystagmus. These data provide strong evidence that alcohol or heavy water entering the labyrinth from the blood decreases or increases the local density of the endolymph. Ethyl alcohol has a specific gravity near 0.8, ~20% lower than endolymph, so any alcohol entering the endolymph from the blood will lower its density and generate a gravity-dependent buoyancy force. The vestibular labyrinth is largely avascular with the critical exception of dense capillary beds in the cristae, utriculus and sacculus. These are the sites where alcohol in the bloodstream enters the endolymph, illustrated in Fig. 6*E* (green arrows) for the LC. When blood alcohol begins to enter the endolymph by diffusion from capillaries, it reduces the density in the cupula and fluid near the crista causing a condition very similar to “light cupula.” Buoyancy-driven deflection of the cupula drives the compensatory VOR, thus leading to positional alcohol nystagmus. Over time, alcohol diffuses throughout the entire labyrinth including the avascular regions remote from the end organs. Once the concentration is uniform, buoyancy is eliminated and the positional component of alcohol nystagmus ceases. Upon metabolizing and clearing alcohol from the blood, the direction of alcohol transport reverses (Fig. 6*F*, blue arrows), slowly removing alcohol from the endolymph. Clearance takes place via capillaries located in the end organs, so it takes considerable time before all alcohol is removed from remote regions of the canal ducts. During this clearance phase, the endolymph remote from the cupula is buoyant, so the force on cupula reverses sign and the direction of alcohol nystagmus reverses, analogous to heavy cupula (Koizuka et al. 1989). Alcohol nystagmus is driven by gravity-dependent inputs from all three canals plus a gravity-independent component putatively arising from the action of alcohol on vestibular neural pathways (Fetter et al. 1999; Haslwanter 2000). The LCs and horizontal VOR are the most susceptible, likely owing to morphology and alcohol transport at multiple end organ sites in addition to interaural alcohol asymmetry.

### Benign Paroxysmal Positional Vertigo

Benign paroxysmal positional vertigo (BPPV) was first described by Robert Bárány in 1921 and is characterized by pathological sensitivity of the semicircular canals to head orientation relative to gravity (Bárány 1921; von Brevern et al. 2015). The condition was further characterized by Dix and Hallpike (1952), who introduced a provocative maneuver in 1952 that is commonly referred to as the “Dix-Hallpike” maneuver and used to evoke PC BPPV. BPPV is caused by the presence of pathological heavy particles inside the membranous labyrinth called *canaliths* (derived from the Latin *canalis* meaning pipe, and the Greek *lithos* meaning stone). The particles are putatively ectopic otoconia that have detached from the macula and moved into a sensitive region of the labyrinth. Although often idiopathic, there are identified risk factors for BPPV including vitamin D deficiency, low bone mineral density, and head trauma (Akin et al. 2017; Chan et al. 2017; Gokler et al. 2018; Rhim 2016; Yang et al. 2018; Yu et al. 2014). Otoconia range in size from less than 1 to 30 µm and are almost threefold denser than endolymph with a specific gravity of ~2.7, (Kniep et al. 2017; Lim 1984; Vibert et al. 2008). Canalith particles can be free to move within the endolymph causing the condition *canalithiasis* or can be adhered to the cupula causing the condition *cupulolithiasis* (Brandt and Steddin 1993; Epley 1992; Hall et al. 1979; Parnes and McClure 1992; Schuknecht 1962). Cupulolithiasis results in a heavy cupula, a condition described above where a change in orientation of the head relative to gravity evokes a response that builds up following the canal time constant (*Eq. 3*) and is maintained as long as the gravitational stimulus is present (*Eq. 6*). This results in sustained inputs to the VOR and sustained slow-phase eye movements. The mechanics of canalithiasis is more complex.

**Fig. 7.**
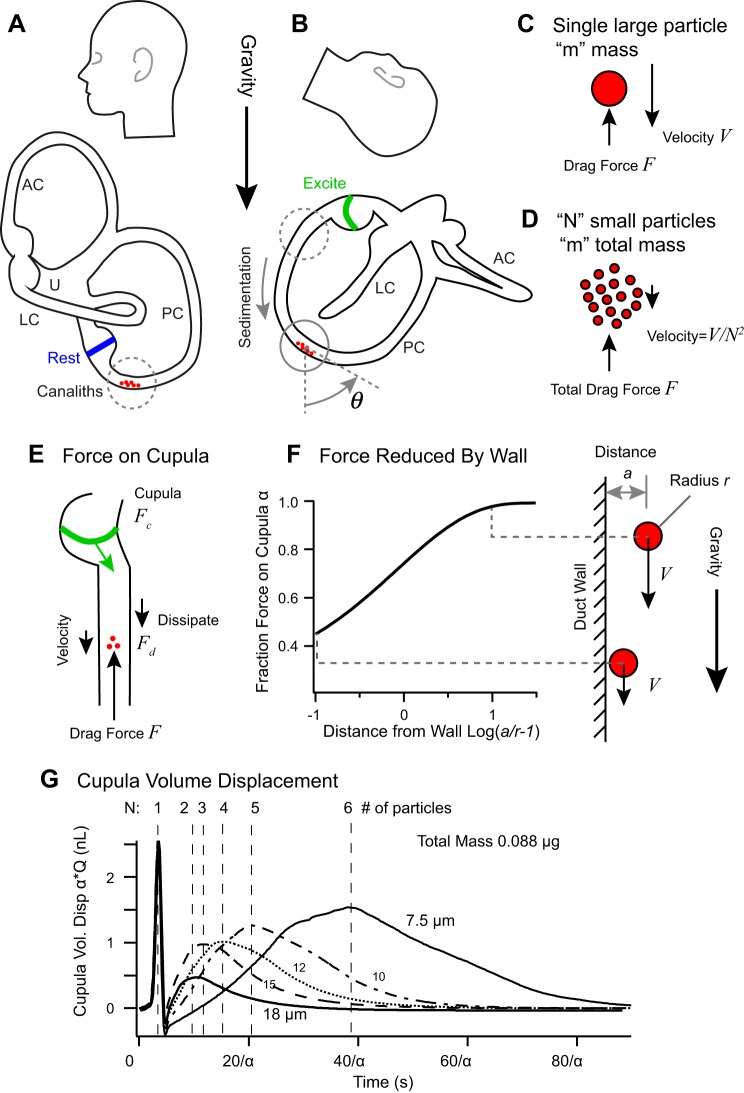
Canalithiasis benign paroxysmal positional vertigo (BPPV). *A*: canalith particles resting at the lowest point in the posterior canal. AC, anterior semicircular canal; PC, posterior canal; LC, lateral canal; U, utriculus. *B*: Dix-Hallpike maneuver positions the canal for particles to sediment ampullofugual down the lumen causing excitatory deflection of the cupula. *C* and *D*: for steady sedimentation, the total drag force balances the gravitational force arising from the total particle mass. *E*: the drag force F generated by the sedimenting canaliths is divided between viscous drag on the lumen of the duct and force on the cupula. *F*: if the particle sediments close to the inside wall, the viscous drag increases and the sedimentation velocity slows. *G*: predicted cupula volume displacements evoked by the Dix-Halpike maneuver for particles of different size but equal total mass. Large particles sediment faster than small particles resulting in shorter latency responses. If particles come to a rest before the cupula has fully deflected, the magnitude of the response will be reduced (specific simulations for α = 1). [*A*, *B*, and *G*: adapted from Rajguru et al. (2004).]

Since the density of canaliths exceeds endolymph, free-floating particles sediment in the lumen of the labyrinth under the action of gravity. Moving particles generate a drag force with endolymph that is partially transferred through the fluid to the cupula and partially dissipated by viscous interaction with the duct wall. While ectopic particles located in the large utricular vestibule have little impact on canal mechanics, particles within a slender duct cause pathological gravity-dependent deflection of the cupula. Sensitivity depends on the location, number, and size of the particles. Ectopic particles can be present in any canal or any ampulla, but the most common form of BPPV afflicts the long arm of the PC as illustrated in Fig. 7*A*. When the head is tilted back, canaliths will sediment ampullofugal (away from the ampulla) deflecting the PC cupula in the excitatory direction (Fig. 7*B*). This increases the discharge rate of PC canal afferent neurons resulting in inappropriate gravity sensitive inputs to the CNS that occur during the time course of sedimentation. Compensatory eye movements reflecting activation of the afflicted canal(s) occur.

The duration and magnitude of sedimentation can be described using simple mechanical principles. With the use of a quasisteady approximation, canalith particles sediment along the canal with a velocity relative to the endolymph of

(10) $$V\approx \frac{\alpha 2r^{2}(\rho_{s}-\rho )g_{s}}{9\mu }$$

where *r* is the particle hydraulic radius, *g*_s_ is the component of gravito-inertial acceleration tangent to the canal lumen, and µ is the endolymph viscosity (House and Honrubia 2003; Rajguru et al. 2004). The parameter α accounts for influence of the canal wall, which slows the particle through viscosity when near the wall (Fig. 7, *C*–*F*) (Chaoui and Feuillebois 2003; Humphrey and Murata 1992; O’Neill and Stewartson 1967). Large particles sediment much faster than small particles, with the velocity scaling as the hydraulic radius squared. The net pressure drop generated by sedimenting canalith(s) is

(11) $$f_{lith}\approx \sum_{1}^{N}\frac{6\pi r\mu V_{c}}{A},$$

where the sum is over all particles in the canal, *V*_c_ is the velocity of each canalith tangent to the centerline, and *A* is the cross-sectional area of the duct at the location of each canalith. *Equations 10* and *11* have been simplified here by assuming angular head rotations are short relative to the sedimentation time and inertial acceleration is small relative to gravitational acceleration (Rajguru et al. 2004). The force per particle is proportional to the particle volume or hydraulic radius cubed. Hence, maximum pressure is dependent on the total mass of all particles in the canal, while sedimentation velocity is proportional the frontal area of a single particle. The sedimentation pressure given by *Eq. 11* drives volume displacement of the cupula and adds to the head acceleration term on the right-hand side of *Eq. 1* (*f* = *f_acc_* + *f_lith_*). To evaluate *f_lith_* numerically, it is necessary to track the position of each particle by integrating the velocity over time while solving *Eq. 1* simultaneously (Rajguru et al. 2004).

The mechanics of sedimentation reveals several important features of canalithiasis BPPV. First, and most important, is that the cross-sectional area containing the particles determines the hydraulic lever responsible for amplifying large-displacement low-force sedimentation in the lumen into a small-displacement large-force deflection of the cupula. As a result of this hydraulic lever, particles sedimenting in the slender lumen of the duct evoke large neural responses, while particles moving in the relatively large utricular vestibule do not. Second, small particles sediment much more slowly than large particles causing nystagmus to last much longer. Third, large particles can sediment to the bottom of the canal faster than the cupula can move (*Eq. 2*), thus resulting in rapid neural responses with a magnitude much smaller than that evoked by a larger number of small particles with equivalent total mass. This is illustrated in Fig. 7*G* for a single 18-µm particle vs. six 7.5-µm particles (curves for α = 1). Finally, the drag force produced by very small particles sedimenting along the wall can be dissipated by viscous interaction with the wall rather than transferring to the cupula (Fig. 7*F*). Based on theory, an infinitely small heavy particle (called a Stokeslet) sedimenting along the surface of a perfectly smooth wall will not evoke mechanical displacement of the cupula, while the same particle sedimenting in the center of the duct will (Squires et al. 2004). In practice, since the epithelial cells lining the membranous labyrinth are not perfectly smooth and since canaliths have real size, neural responses are evoked even if the particles are sliding or rolling along the wall (Rajguru and Rabbitt 2007). Nevertheless, neural responses will be reduced if the particles are very small and sediment in close vicinity to a smooth wall, an effect estimated by the scaling factor α on both axes in Fig. 7*G* (specific simulations use α = 1). This effect can significantly slow the sedimentation velocity and reduce the magnitude of evoked responses, thus making clinical repositioning of small canaliths more difficult than large canaliths.

**Fig. 8.**
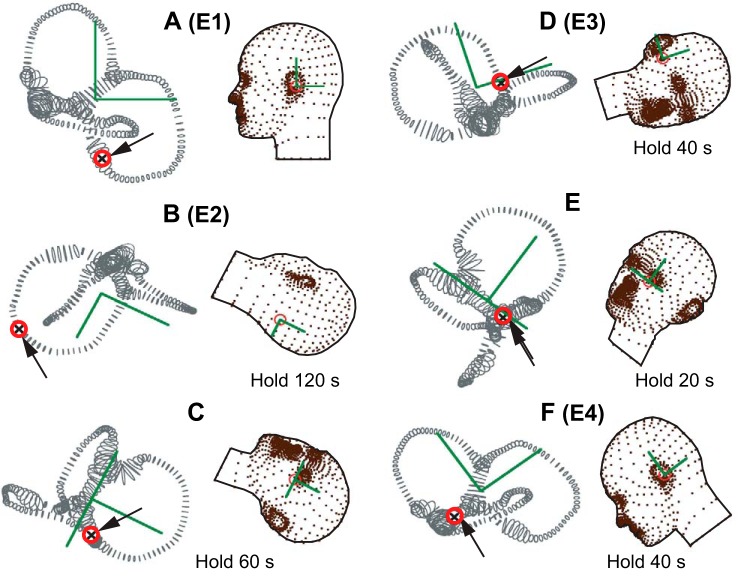
Modified Epley canalith repositioning procedure. Sequence of 6 head positions (*A*–*F*) designed to move canaliths (arrows) from the long arm of the posterior canal in the right ear to the utricular vestibule. Hold times shown in each position are estimates based on relatively small, slowly sedimenting, canaliths. *A*: initial seated position (Epley 1). *B*: rotation into the Dix-Hallpike head hanging position toward the right ear to sediment particles as shown in Fig. 7. (Epley 2). *C*: rotation toward the left ear to sediment particles toward the common crus. *D*: head held up rotated left to move particles into crus avoiding conversion to the anterior canal (Modified Epley 3). *E*: return toward upright position with particles sedimenting down the common crus. *F*: head tipped forward to move particles to nasal region of the utricular vestibule (Epley 4). [Based on Rajguru et al. (2004).]

Canalithiasis BPPV can be treated by moving the particles to the utricular vestibule using a canalith repositioning procedure (CRP). One of the most successful CRPs was first introduced by J. Epley to treat BPPV of the PC (Epley 1992). A modified version of the “Epley CRP” consisting of six head positions is shown in Fig. 8 (Rajguru et al. 2004). This particular CRP moves particles from the long arm of the PC to the utricular vestibule. When the subject is sitting in the upright position, PC canaliths will sediment into the lowest section of the PC. Turning the head toward the afflicted ear and then hanging the head back (Fig. 8*B*: Dix-Hallpike position, or Epley position 2) will cause canalith sedimentation toward the common crus. The head needs to be held in this position sufficiently long for sedimentation to complete, which, as described above, takes much longer for small particles. Progress can usually be assessed by monitoring slow-phase eye movements. While still hanging, the head is rotated toward the opposite ear (Fig. 8*C*) to drive the particles further toward the common crus. The head is then tipped up and rotated further with the afflicted ear up (Fig. 8*D*) to allow canaliths to sediment into the common crus. It is important to keep the head tipped up to prevent particles from entering the anterior canal and converting to AC BPPV. Once particles are in the common crus, the head is tipped further up (Fig. 8*E*) to allow sedimentation into the utricular vestibule and then tipped forward (Fig. 8*F*) to move the particles toward the utriculus. Once in the utricular vestibule, pathological gravity sensitivity no longer occurs because the hydraulic lever is eliminated.

A variety of CRPs have been devised to reposition canaliths from almost any sensitive location in the labyrinth. The clinical challenge is to determine the initial location of the canaliths based on observation of gravity-dependent slow-phase eye movements and to perform the appropriate CRP with effective orientations relative to gravity and sufficient hold times (Helminski et al. 2010). Although CRPs are highly successful in classical presentations (Akin et al. 2017), CRPs can fail in some cases; for example, if canaliths are not truly free to sediment but instead are tethered by macromolecules to the membranous labyrinth, if the lumen is partially occluded to prevent canaliths from sedimenting past a certain location in the canal, or if canalith particles are pervasive throughout multiple sensitive regions of the labyrinth.

### Perilymphatic Fistula or Dehiscence of the Bone

Prominent 19th century scientists argued that the semicircular canals might be sensitive to air-conducted sound and potentially could play a role in directional hearing (Camis 1930). Although we now know the canals are highly selective to angular motion and normally protected from stimulation by air-conducted sound, early experiments by Deetjen (1899) seemed to support the acoustic sensitivity hypothesis. Deetjen (1899) introduced aluminum powder into the perilymph and visualized both vibration and continuous flow of the fluid in response to sound. According to Camis (1930), Deetjen (1899) conjectured that forces and fluid motion in the perilymph might be transmitted through the membranous labyrinth to the endolymph providing a route for transduction by the crista, but Deetjen also noted evidence that mammalian vestibular organs are unlikely to be involved in auditory perception. Tullio (1929) demonstrated 30 yr later that the semicircular canals are normally insensitive to sound but can become pathologically sensitive if a fistula is opened in the bony labyrinth. The experiments of Deetjen (1899) were correct, but the sound-evoked perilymph motion he observed was pathological and occurred only after opening the bony labyrinth. Indeed, individuals with a perilymphatic fistula or dehiscence of the bone enclosing the labyrinth can experience pathological canal sensitivity to sound: a condition called Tullio phenomena*.* A fistula or dehiscence can also introduce other symptoms including conductive hearing loss, pressure sensitivity, and increased vestibular sensitivity to bone-conducted vibration (Arts et al. 2009; Mikulec et al. 2004; Minor et al. 1998; Songer and Rosowski 2010; Ward et al. 2017).

**Fig. 9.**
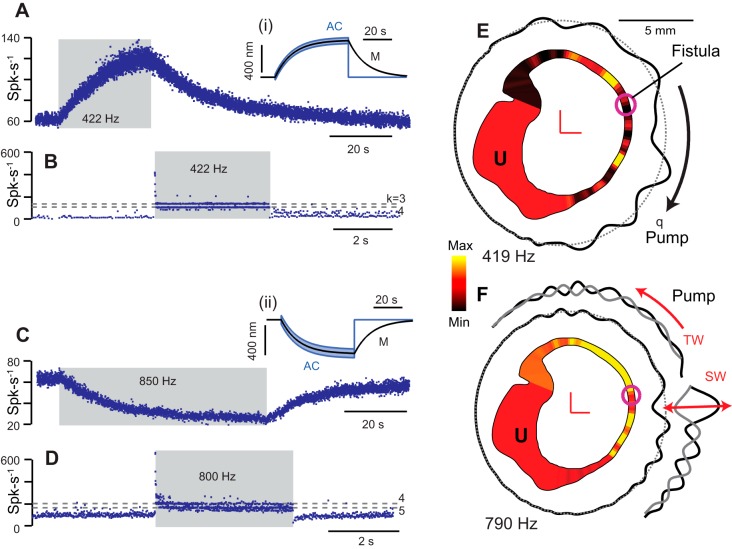
Auditory frequency responses with dehiscence or fistula. *A*–*D*: single-unit afferent neuron responses to auditory frequency stimuli in an animal model of dehiscence. *A* and *B*: example afferent neuron excited by 422-Hz auditory frequency stimulation through nonlinear endolymph pumping causing slow buildup of discharge rate (*A*) and another afferent in the same animal phase locking action potential firing to the auditory frequency stimulus at winding ratios (*B*), *k* = 3,4,5. *Insets* *i* and *ii*: illustration of the slow component of cupula displacement (M, black) evoked by the auditory frequency driven endolymph pumping and cycle by cycle vibration around the deflected position (AC, blue). *C* and *D*: example afferent neuron inhibited by endolymph pumping for a 850-Hz stimulus (*C*) and another afferent in the same animal phase locking action potential at winding ratios (*D*), *k* = 4.5. *E* and *F*: computational model of a human semicircular canal showing slowly developing pressure distribution (yellow: high; red: zero; and black: low) evoked by auditory frequency stimulation at 419 Hz (*E*) and 790 Hz (*F*). Waves travel along the membranous labyrinth away from the site of the fistula causing vibration of hair bundles at the stimulus frequency and pumping of endolymph in either direction, ampullofugal for 419 Hz (*E*) and ampullopetal for 790 Hz (*F*). SW, standing waves; TW, traveling waves. [Based on Iversen et al. (2018).]

Semicircular canal afferent nerve responses to auditory frequency stimuli in animal models of canal dehiscence syndrome have revealed two characteristic types of responses: *1*) increases or decreases in action potential discharge rate that build up with the time constant of the canal, and *2*) entrainment of the discharge rate to the auditory frequency stimulus with action potentials occurring at a precise phase relative to the stimulus (Carey et al. 2004; Iversen et al. 2018). In mammals, neurons of the first type typically have regular interspike intervals at rest, while neurons with of the second type are calyx bearing and have irregular interspike intervals at rest (Curthoys et al. 2016). Example single-unit afferent neuron responses in the toadfish model of canal dehiscence syndrome are shown in response to auditory frequency stimulation at 422 Hz (Fig. 9, *A* and *B*) and at 800 Hz (Fig. 9, *C* and *D*). Units shown in Fig. 9, *A* and *C*, do not phase lock to the auditory frequency stimulus and modulate their discharge rate in either the excitatory or inhibitory direction depending on frequency, while units shown in Fig. 9, *B* and *D*, phase lock discharge and always respond as excitatory. The same type of neural responses have been observed in a mammalian model of canal dehiscence syndrome (Carey et al. 2004). Both types of auditory frequency responses arise directly from pathological hair bundle displacements in the compromised labyrinth.

Auditory frequency mechanics of semicircular canals is complex and nonlinear, involving the interaction of perilymph, endolymph, and the thin deformable membrane separating the two. The sensory organs themselves also play a role due to their location, morphology, and mechanical properties. A complete analysis of the deformable three-dimensional apparatus has not yet appeared in the literature, but some insight can be gained from analysis of simplified deformable canals. Much like the cochlea, the semicircular canals consist of two fluids, endolymph and perilymph, separated by a flexible partition. The partition deforms if there is a pressure gradient across it, thus driving fluid displacement and ultimately leading to displacement of sensory hair bundles. The two fluids have density responsible for storing and releasing kinetic energy and viscosity responsible for dissipating power. The membrane has elasticity responsible for storing and releasing potential energy. The interaction between kinetic energy and potential energy combined with viscosity results in a dispersive wave equation (Grieser et al. 2016; Iversen and Rabbitt 2017), similar to the equations describing pulsatile wave propagation in arteries, and traveling wave propagation in the cochlea (Atabek and Lew 1966; Fletcher 1951; Fung 1990). Under normal conditions, the canals are protected from auditory frequency stimulation by the bony enclosure and hence waves are not generated in the canals. This protection is lost if the bony labyrinth is compromised.

A fistula or dehiscence of the bone generates a pressure relief point that is referred to as a “third window” augmenting the oval and round windows. Part of the sound energy entering the inner ear at the oval window is diverted toward the dehiscence, thus accounting for the conductive hearing loss (Songer and Rosowski 2010). This diverted sound energy generates a pressure gradient in the vestibular apparatus that can excite traveling waves along the membranous labyrinth (Grieser et al. 2016; Iversen et al. 2018). Conservation of mass requires the perilymph volume displacement entering the affected canal near the utricular vestibule to be balanced by an equal volume displacement at the fistula. Since the utricular vestibule is much larger than the fistula, the perilymph velocity at the fistula is much larger than near the utricle. This generates a large pressure gradient acting across the membranous labyrinth between endolymph and perilymph at the site of the fistula. As a result, traveling waves are generated at the fistula and propagate toward the utricle (Iversen et al. 2018). The direction of propagation is somewhat counterintuitive, because the waves travel toward the sound source rather than toward the fistula. This is illustrated based on the model of Iversen and Rabbitt (2017) in Fig. 9, *E* and *F*, showing build-up of transmembrane pressure (color scale: yellow, is max, red is 0, black is min) and traveling waves (black wavy curves) for auditory frequency stimuli at 419 and 790 Hz. Waves travel away from the site of the dehiscence and pump fluid in both directions generating pressure across the cupula that builds up following the slow time constant of the cupula.

In addition to endolymph pumping, waves traveling ampullopetal from the site of the fistula pass through the ampulla thus vibrating sensory hair bundles at the auditory stimulus frequency. This vibration modulates MET currents leading to transmitter release and action potential discharge locked in step with the vibration. In sensitive irregularly discharging neurons, action potentials are evoked with a specific delay relative to the bundle displacement resulting in phase-locked firing occurring at every *k*^th^ cycle of the stimulus (*k* = 1,2,3… is the winding ratio, e.g., Fig. 9, *B* and *D*) (Curthoys et al. 2016). In addition to the cycle-by-cycle vibration, traveling waves pump the endolymph due to nonlinear interaction of the fluid and the undulating membrane (Grieser et al. 2016; Iversen et al. 2018). Since waves travel in both directions away from the fistula, the net direction of pumping depends on which wave dominates. Frequency-dependent reflection of the waves can generate standing waves in one direction that do not pump fluid (Fig. 9*F*), but traveling waves in the other direction that do pump fluid (Fig. 9*F*). The net result is a traveling wave pump that drives endolymph in a frequency-dependent way to slowly deflect the cupula in an excitatory or inhibitory direction, evoking responses like the examples shown in Fig. 9, *A* and *C*. Auditory frequency endolymph pumping has been directly observed in the semicircular canals of an animal model using particle imaging velocimetry (Iversen et al. 2018). This is a nonlinear effect with similarities to a classical Liebau valveless pump (Liebau 1954; Thomann 1978). The only known treatments are surgical repair of the fistula or canal plugging (Ward et al. 2017).

### Additional Consequences of Membranous Labyrinth Compliance

Encasement in rigid bone normally protects the membranous labyrinth from pressure gradients that would otherwise occur. Owing to complete enclosure, linear acceleration generates equal pressures in the endolymph and perilymph thus eliminating any pressure gradient across the membrane and rendering the canals insensitive to linear acceleration. Pressure applied to the oval window generates nearly uniform pressure in canal perilymph and endolymph (Fig. 1*A*, blue), again eliminating any sound or ear canal pressure induced deformation of the membranous labyrinth. Opening the bony labyrinth breaks the balance and introduces stimulus-evoked transmembrane pressure gradients that normally would not exist. Since the membranous labyrinth is thin, even small pressure gradients can deform it. The damaged canal becomes sensitive to sound, vibration, static pressure, blood pressure, and linear acceleration. However, a fistula or dehiscence are not the only conditions that generate important transmembrane pressure gradients.

In the intact labyrinth, pressure gradients evoked in the endolymph and perilymph by sinusoidal angular head movements are not identical to each other simply because of differences in fluid mechanics associated with differing morphologies of the two ducts. The pressure difference increases with stimulus frequency as the inertial force increases, eventually reaching a point where the membranous labyrinth deforms. This is likely to occur at frequencies above 6 Hz in humans, where perilymph fluid mechanics contributes to deform the membrane, thus driving endolymph displacement and increasing canal sensitivity to angular head rotation (Fig. 4*F*, solid curve) (Iversen and Rabbitt 2017). It is not clear if the increased high-frequency sensitivity provided by this mechanism offers an advantage to the animal or not. Further evidence for membranous labyrinth deformation at high frequencies is the fact that surgically plugged semicircular canals continue to respond to high-frequency sinusoidal head rotation. Surgical plugging of a rigid canal would completely eliminate endolymph displacement and thereby completely eliminate sensitivity to angular motion stimulation. This is almost true for low-frequency sinusoidal oscillations where canal plugging attenuates afferent responses to head rotation >100-fold as if the canal time constant (*Eq. 3*) has been decreased dramatically (Sadeghi et al. 2009). Plugging often completely fails to eliminate responses above 6 Hz due to high inertial forces and pressure (Hess et al. 2000; Rabbitt et al. 1999). Sensitivity persists at high frequencies because stimulus-evoked pressure differences between endolymph and perilymph deform the membranous labyrinth, thus causing cupula deflection even when the canal is plugged. Another example is provided by afferent responses to episodic increases in endolymph pressure, pressure possibly generated by a transient osmotic imbalance between the inner ear fluids. It has been shown in an animal model that a hydrostatic pressure increase in endolymph distends the ampulla and thereby evokes changes in the discharge rate of afferent neurons through hair bundle deflections (Yamauchi et al. 2002). It is currently not known if mechanical sensitivity to transient transmembrane pressure changes might be related to episodic attacks of vertigo in Ménière’s disease or other conditions (Gurkov et al. 2016; Salt and Plontke 2010).

## CONCLUSIONS

Gross semicircular canal morphology is remarkably conserved through phylogenesis, with only relatively minor variations appearing across amniotes. Although there are significant differences in hair cells and synapses between species, the fundamental biomechanical contributions to canal selectivity to angular motion, frequency-dependent sensitivity, directional coding, and temporal coding are universal. The origins these sensory characteristics can be quantified using first principles of mechanics. Decomposition of 3D movements into three vectoral components, one carried by each canal nerve, is the result of macromechanics (Fig. 1). The lower corner frequency defining the transition from angular acceleration sensitivity to angular velocity sensitivity and wave propagation responsible for sound and vibration sensitivity also result directly from macromechanics (Fig. 4). Mechanical principles can be applied to understand substrates of a class of common vestibular disorders and in some cases can be used to devise effective therapeutic treatments. Future optimization of CRPs and other mechanical procedures therefore might benefit from subject-specific biomechanical analysis drawing from imaging modalities capable of resolving detailed morphology of the patient's membranous labyrinth. On a finer scale, micro- and nanomechanics of long hair bundles and mechanical aspects of transduction have received relatively little attention to date in the semicircular canals and represent areas where current understanding is incomplete.

## GRANTS

This work was supported by National Institute of Deafness and Other Communications Disorders Grant R01-DC-006685.

## DISCLOSURES

No conflicts of interest, financial or otherwise, are declared by the authors.

## AUTHOR CONTRIBUTIONS

R.D.R. conceived and designed research; R.D.R. performed experiments; R.D.R. analyzed data; R.D.R. interpreted results of experiments; R.D.R. prepared figures; R.D.R. drafted manuscript; R.D.R. edited and revised manuscript; R.D.R. approved final version of manuscript.
